# A Retrospective Chart Review Evaluating the Changes in Depressive Symptoms in Adult Patients With Attention-Deficit/Hyperactivity Disorder (ADHD) With the Treatment of the Underlying ADHD

**DOI:** 10.7759/cureus.57473

**Published:** 2024-04-02

**Authors:** Ushna Shamoon, Jude K Des Bordes, Shira Goldstein

**Affiliations:** 1 Department of Family and Community Medicine, University of Texas Health Science Center at Houston McGovern Medical School, Houston, USA

**Keywords:** attention-deficit/hyperactivity disorder, adult, integrated behavioral health, depression, adhd

## Abstract

Introduction: Adult attention-deficit/hyperactivity disorder (ADHD) represents a significant public health burden. ADHD is often comorbid with many other psychiatric disorders, with a high co-occurrence with depression. However, there is a paucity in our understanding of the potential impact of treating patients’ ADHD on their depressive symptoms. The primary objective of this study was to assess the effect of treating adult ADHD on comorbid depressive symptoms without directly administering treatment for depression in an integrated behavioral health clinic in the primary care setting.

Methods: We performed a retrospective chart review between April 2021 and May 2022 on adult patients treated in the Primary Care Adult Integrated Behavioral Health Clinic at an urban family medicine residency clinic. For patients with ADHD, we administered the Adult ADHD Self-Report Scale (ASRS-v1.1) to serve as a marker of ADHD symptom burden and the nine-item Patient Health Questionnaire (PHQ-9) to serve as a marker of depressive symptom burden. We administered the questionnaires prior to initiating ADHD treatment and again at the three-month follow-up visit. The ADHD treatment included pharmacotherapy and brief psychological interventions targeted at ADHD. We compared the ASRS scores and PHQ-9 scores at baseline and after three months to determine whether ADHD treatment had any impact on PHQ-9 scores.

Results: At baseline, the average ASRS score was 11.3 and the average PHQ-9 score was 8.25. Comparing scores after three months of intervention to the initial scores, our preliminary results demonstrated a trend of improvement in both ASRS and PHQ-9 scores. A total of 75% (n=24/32) of the patients had an improvement in ASRS scores, and 56.7% (n=17/30) of the patients had an improvement in PHQ-9 scores at three months. At three months, there was a decline in PHQ-9 scores with a decrease in ASRS scores following treatment.

Conclusion: Our preliminary results suggest that integrated behavioral health treatment of ADHD using a combination of pharmacological and non-pharmacological interventions may play a role in improving comorbid depressive symptoms.

## Introduction

Adult attention-deficit/hyperactivity disorder (ADHD) is a prevalent psychiatric disorder among the adult population, affecting about 366 million adults, characterized by inattention, impulsivity, and hyperactivity [1,2]. ADHD is often comorbid with many other psychiatric disorders, with a high co-occurrence with depression [3,4]. As such, it is vital that we not only continue to develop and improve our diagnostic and therapeutic strategies for adult ADHD but also gain a better understanding of how our current treatment plans for ADHD impact secondary illnesses. Current guidelines for the treatment of ADHD in adult populations recommend a combination of medication and ADHD-targeted behavioral therapy [5,6]. However, there is a paucity of literature that demonstrates the effect of ADHD treatment on comorbid depression [7]. 

Due to the high comorbidity of depression with ADHD and the persistence of ADHD into adulthood, this study evaluates the effect of an integrated behavioral health clinic’s treatment of ADHD on comorbid depressive symptoms [8]. We hypothesize that an integrated behavioral health approach offering a combination of pharmacological and psychological treatment of ADHD will improve comorbid depressive symptoms in adult patients with ADHD. Specifically, based on a study by Houghton et al. (2017), which demonstrated the improvement of comorbid anxiety with the treatment of ADHD, we hypothesize that with the Adult ADHD Self-Report Scale (ASRS-v1.1) (see Appendices) and the nine-item Patient Health Questionnaire (PHQ-9) (see Appendices) serving as markers of ADHD symptoms and depressive symptoms, respectively, both will improve (decrease) with the treatment of ADHD using pharmacotherapy and brief psychological interventions [9]. 

This study was presented as a poster presentation at the 2023 Society of Teachers of Family Medicine Annual Spring Conference in April 2023. 

## Materials and methods

Study design

This retrospective quantitative pilot study was conducted at the University of Texas (UT) Physicians Family Medicine Clinic - Texas Medical Center, Houston, Texas, United States, from April 2021 to June 2022. This study used data available after author SG recently started the Primary Care Adult Integrated Behavioral Health Clinic in the Family Medicine Office, intending to use the initial information to further improve the treatment of patients in this clinic.

Ethical considerations

As the standard of care was adhered to while treating the patients with ADHD, only delivered in a novel way, ethical concerns were limited. This research was approved by the UTHealth Houston Institutional Review Board (HSC-MS-22-0074).

Study criteria

Our study sample included adult patients at an urban family medicine clinic internally referred by the family physicians to the Primary Care Adult Integrated Behavioral Health Clinic to assess for and treat adult ADHD when appropriate. A family physician and a psychologist both with expertise in diagnosing and treating ADHD co-facilitate this clinic which is housed in the Family Medicine Office. Patients included in this study had either a prior diagnosis of ADHD but were not receiving treatment or were newly diagnosed with ADHD. New diagnoses of ADHD were made in this clinic via a structured psychological interview of the patients often with input from a loved one, the ADHD symptom checklist via ASRS, screening for other psychiatric disorders, and a comprehensive medical history and physical exam. 

Inclusion criteria for this study included adults aged 18 and over with a diagnosis of ADHD who were seen at the clinic and who were not currently receiving treatment but wished to initiate treatment.

Exclusion criteria included patients without a diagnosis of ADHD, patients with contraindications to stimulants or serotonin and norepinephrine reuptake inhibitor (SNRI) treatment, or who did not desire to initiate pharmacotherapy.

Procedure

The clinic operates for half a day every other week and is physically located in the UT Physicians Family Medicine Clinic - Texas Medical Center. The patient, family physician, and psychologist have a combined visit. During the 40-minute visit, following history taking and physical exam, if the patient is diagnosed with ADHD, the family physician recommends medication therapy if appropriate, and the psychologist leads a brief psychological intervention consisting of ADHD coaching, behavioral therapy, and psychoeducation [10]. 

Medication management of ADHD for those included in this study included treatment with stimulant therapy or SNRIs, which are FDA-approved for the treatment of ADHD and are the standard of care.

Assessments

At the initial visit and all subsequent visits, all patients completed an ASRS-v1.1 form with a possible score range of 0-18 (18 representing the highest symptom burden) and a PHQ-9 form with a possible score range of 0-27 (27 representing the highest symptom burden) [11,12]. The patients with ADHD had clinic follow-ups monthly during the first three months to assess medication tolerability and efficacy, and then they were seen every three months. We used the validated questionnaires ASRS and PHQ-9 as measures of ADHD and depression, respectively [13,14].

Sample size calculation

The sample size was based on the number of patients who met the above-required criteria and completed either the ASRS or the PHQ-9 questionnaire during their initial clinic visit as well as during their three-month follow-up visit.

Data collection

A retrospective chart review was performed in this study because it allowed the authors to evaluate for a potential change in depressive symptomatology, which may require time to demonstrate improvement. The results of the scores from baseline and after three months of intervention for patients seen in the clinic between April 2021 and May 2022 were reviewed. We also collected demographic information (age, sex, and race). Race was categorized as White and other, if non-Caucasian.

Statistical analysis

We performed a descriptive analysis of the baseline data summarizing categorical variables using frequencies and percentages, and continuous variables as means and standard deviations. We described the participants by demography (age, sex, and race). We calculated the mean scores of the ASRS and PHQ-9 at baseline and at three months and assessed significance using the Student's T-test. Patients were considered to have improved if their follow-up scores were lower than baseline, unchanged if the scores were at baseline, and worse if the follow-up scores were higher than baseline. We then compared the association of improvement in ASRS scores with improvement in PHQ-9 scores at three months using a Chi-square test to determine whether ADHD treatment had any effect on PHQ-9 scores.

## Results

A total of 56 patients met the criteria for our study. Our analysis included 26 patients of the original 56 patients who came to the monthly visits and completed the PHQ-9 and ASRS questionnaires at three months. A total of 30 patients were lost to follow-up, did not continue treatment, or did not show up to one of the visits. The age of participants ranged from 18 to 67 years with a mean age of 35.7 ± 12.5 years. Nearly 62% were female and 58% were White. The average ASRS score at baseline prior to intervention was 11.8 ± 3.93 (out of a total possible score of 18) and the average ASRS score at three months was 8.46 ± 4.14 (p<0.05). For the PHQ-9, the average score at baseline was 8.88 ± 4.69 (out of a total possible score of 27) and at the three-month visit, the average score was 7.58 ± 6.65 (p=0.42). We found that at the three-month evaluation, 76.9% (20/26) of patients had an improvement in (reduction in score) ASRS scores, and 57.7% (15/26) had an improvement in PHQ-9 scores [1-3.] Therefore, we found that improvement in ASRS scores was associated with improvement in PHQ-9 scores at three months, Pearson's χ^2^=5.38, p=0.02.

Table 1 is a summary of the participant characteristics.

**Table 1:** Characteristics of study participants (N=26) SD: standard deviation; ASRS: Adult ADHD Self-Report Scale; PHQ-9: nine-item Patient Health Questionnaire

Characteristic	Quantity
Age (mean ± SD)	35.7 ± 12.5
Race (number, %)	
White	15 (57.7)
Other	11 (42.3)
Sex (number, %)	
Male	10 (38.5)
Female	16 (61.5)
ASRS score (mean ± SD)	
Baseline	11.8 ± 3.9
Three months	8.46 ± 4.14
PHQ-9 score (mean ± SD)	
Baseline	8.9 ± 4.7
Three months	7.58 ± 6.7

## Discussion

We undertook a review of medical records to determine if effective treatment of ADHD resulted in a decrease in patients’ self-reported depressive symptoms. Our results showed that the patients who followed up at the Primary Care Adult Integrated Behavioral Health Clinic at three months generally saw some improvement in their ADHD and depressive symptoms.

The aim of this study was to determine if an integrated behavioral health clinic in a family medicine office providing a combination of pharmacological and non-pharmacological interventions for the treatment of ADHD results in improvement in comorbid depressive symptoms. Our preliminary results suggest that there is a significant association between improvement in ADHD symptoms and improvement in depressive symptoms when treating adults for ADHD. With these results, other programs and institutions managing adulthood ADHD can begin to monitor secondary illness symptoms in order to ultimately have a more well-rounded treatment plan for adults with ADHD that takes into account secondary illnesses and their associated symptoms. Unfortunately, as many patients with ADHD and other behavioral health disorders struggle with follow-up, obtaining these data may be challenging [15]. Unlike medical treatment for depression which may take weeks to months to begin to improve depressive symptomatology, medical treatment for ADHD allows for rapid symptomatic relief. Therefore, it makes sense that while patients demonstrate improvement in ADHD symptoms within the three-month period, that is not enough time to maximally capture improvement in their depressive symptoms. 

The full impact of comorbid psychopathologies on ADHD treatment is still being studied. For example, adolescents with ADHD and comorbid anxiety have decreased clinical response to ADHD pharmacologic therapy alone as compared to those adolescents with only ADHD [16]. However, when treated with a combination of pharmacotherapy and non-pharmacologic interventions like cognitive behavioral therapy (CBT), not only was there an improvement in ADHD symptoms but in comorbid anxiety as well [10,17,18]. Our study uniquely explored depressive symptoms in the setting of ADHD and found preliminary results suggesting improvement in comorbid depressive symptoms with an integrated behavioral health approach to ADHD treatment. Additionally, although improvement in comorbid mood disorders with the treatment of ADHD has been demonstrated in the adolescent population, our study may suggest the same to be true in adult populations as well [16].

The number of patients within this study was our primary limitation; thus, the study was not powered for significance. Since many patients missed the three-month follow-up visit, it is possible that our limited sample was made of motivated patients who were determined to see an improvement in their condition. Additional longitudinal research monitoring the long-term effects of ADHD treatment on secondary illnesses is needed, and we are in the process of collecting these data. Another limitation of this study is that despite the validity of the ASRS and PHQ-9 screeners in detecting ADHD and depression symptom burden, respectively, on a day-to-day basis, there is variability in scores due to exacerbating life stressors occurring in patients' lives [19,20]. For this reason, we are currently developing a qualitative study to supplement our work to provide a more complete understanding of patient symptom burden.

## Conclusions

Adult ADHD represents a significant public health burden. This study suggests that a primary care integrated behavioral health clinic may be a successful way to treat depressive symptoms in adults with ADHD. This is important because while current guidelines recommend treating adult ADHD with a combination of medication and CBT, unfortunately, CBT is often not accessed due to patient and system barriers to participating in therapy. Additionally, given the national shortage of psychiatrists, the burden of treating adult ADHD and mood disorders is on the primary care setting. 

The relationship between ADHD and its comorbid psychopathologies is still being explored. As there is currently no standard protocol for the diagnosis and monitoring of secondary mood disorders in ADHD and the effects of these treatment modalities on secondary illnesses are still unclear, our study proposes the use of the integrated behavioral health clinic model to administer combination ADHD treatment and suggests that it may improve comorbid depressive symptoms in adult populations in the primary care setting.

## Figures and Tables

**Figure 1:**
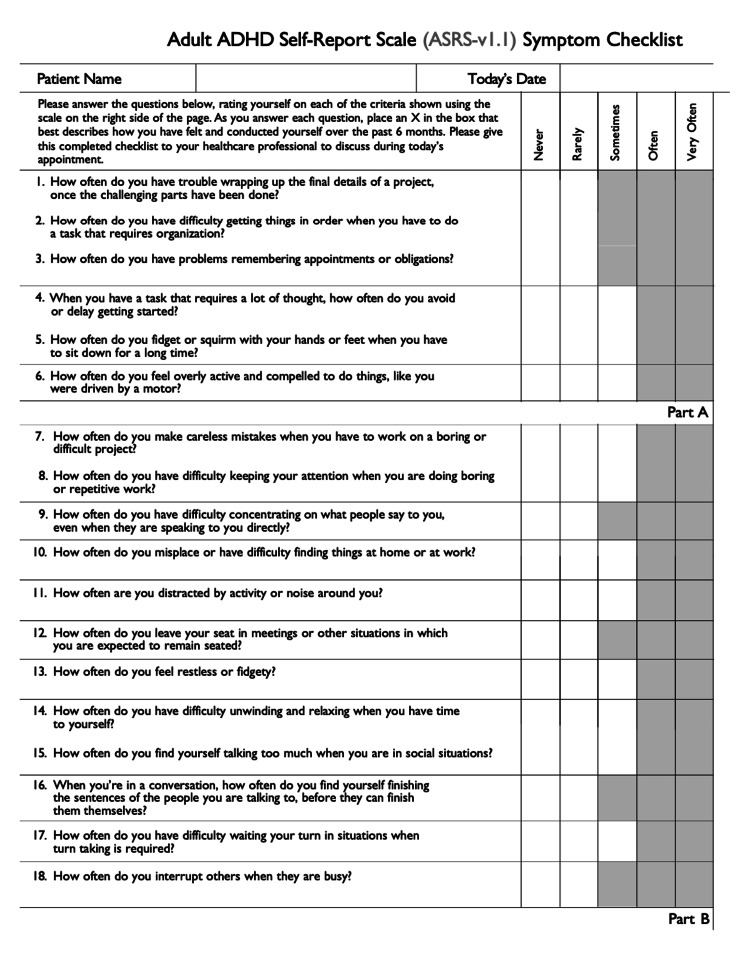
ASRS-v1.1 questionnaire

**Figure 2:**
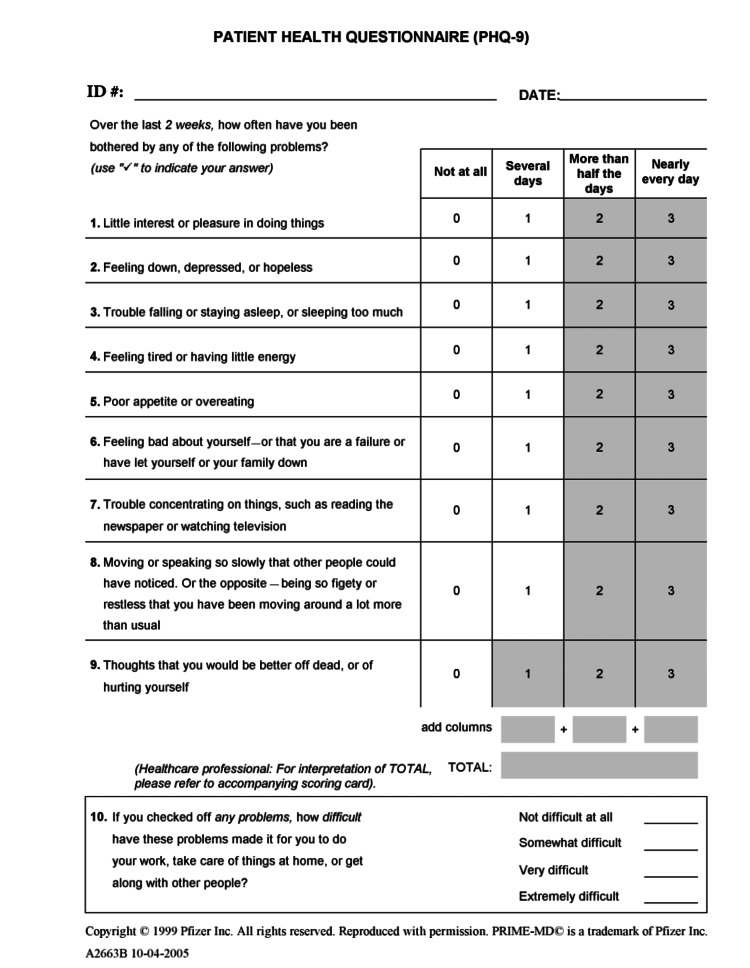
PHQ-9 questionnaire
